# The Sound Produced by a Dripping Tap is Driven by Resonant Oscillations of an Entrapped Air Bubble

**DOI:** 10.1038/s41598-018-27913-0

**Published:** 2018-06-22

**Authors:** Samuel Phillips, Anurag Agarwal, Peter Jordan

**Affiliations:** 1 Emmanuel College, Cambridge, CB2 3AP UK; 2 Department of Engineering, University of Cambridge, Cambridge, CB2 1PZ UK; 3 PPRIME Institute, CNRS - University of Poitiers - ENSMA, Poitiers, France

## Abstract

This paper details an investigation into the characteristic ‘plink’ sound produced by water droplets impacting a liquid surface, such as those falling from a dripping tap. Modern high-speed video and audio capture techniques have been applied to this problem for the first time. Previous literature investigating the underwater sound produced has been validated, with the key sound producing feature both above and below the water confirmed to be the entrainment of a small underwater air bubble. Recorded sound frequencies have been shown to align with the theoretical natural oscillation frequency of the entrained bubble, confirming this to be the driver of the characteristic ‘plink’ sound. For the first time these oscillations of the entrained bubble have been directly observed on video footage. An investigation into the effect of underwater reverberation showed that the airborne sound field is not simply the underwater field propagating through the water-air interface, as had previously been assumed. An alternative hypothesis is that the oscillating bubble induces oscillations of the water surface itself, giving a more efficient mechanism by which the underwater bubble drives the airborne sound field. A model for this new hypothesis produces good agreement with experimental data.

## Introduction

The characteristic ‘plink’ produced by water droplets falling from a dripping tap is universally recognisable and has been a source of scientific curiosity for over a century. Worthington’s 1908 publication “A study of splashes”^1^ contains the earliest known photographs of such drop impacts, showing the formation of a surface cavity which recoils, resulting in a rising column of liquid. This initial study focussed on the fluid mechanics of a drop’s impact, but interest soon turned to the characteristic sound it produced. Around 1920 a number of researchers applied themselves to the problem, with Mallock^2^ and Paget^3^ both citing resonance inside the air cavity as the most likely mechanism behind the sound, whilst Jones^4^ and Raman and Dey^5^ found that there appeared to be a minimum drop height below which no sound was produced. These studies also noted that the drop impact resulted in a small air bubble being trapped underwater, and in 1933 Minnaert^6^, considering the sounds of running water, derived a formula for the natural oscillation frequency of such a bubble, suggesting it as a possible mechanism behind the distinctive ‘plink’ with his closing comment: *“It remains to investigate* […] *if the sounds of falling drops cannot have the same origin as the bubble sounds.”* Richardson^7^ was the first to put this new theory to the test by investigating solid spheres entering water, as these impacts also result in the entrapment of a small air bubble (Narayan^8^, among others, had already noted the similarity of the sound produced by spheres entering water to that produced by drops impacting a liquid surface). Richardson found that the frequencies recorded did scale as suggested by Minnaert’s formula.

Franz’s 1959 paper “Splashes as sources of sound in liquids”^9^ was the first in depth study into the sounds produced by drop impacts. Using a series of photographs Franz highlighted four key features of each impact: the initial contact; the formation of a cavity; the entrainment of an air bubble; and the recoil of the surface producing a vertical jet. Synchronised hydrophone recordings revealed the initial contact and bubble entrainment to be the key sound-generating stages, with a short pulse produced by the impact sound, followed by a delay as the crater forms, before a more significant wave packet is initiated after the bubble is entrained. Due to the low frame rate it was difficult to accurately associate physical events to the generation of the sound field.

Further studies into the fluid mechanics of splashes persisted, including work by Macklin and Metaxas^10^ among others, but little interest was shown in the sound-production mechanism until the late 1980s when it was suggested that the distinctive underwater sound signature of a drop impact could allow a simple hydrophone to be used to measure oceanic rainfall. This required a relationship between the size of a raindrop and the frequency of the sound it produced to be found, and led to extensive research into the underwater sound generated by individual drop impacts. Pumphrey *et al*.^11^ confirmed Franz’s earlier findings on the mechanism and key sound producing features, as well as noting that for a given drop diameter the underwater bubble only formed within a certain range of impact velocities, naming this the region of ‘regular entrainment’. Kurgan^12^ arrived at the same conclusions independently, whilst Medwin *et al*.^13^ looked into the effect of oblique incidences on bubble entrainment. These experimental findings were supplemented by the analytical work of Oquz and Prosperetti^14^, Guo and Ffowcs-Williams^15^ and Longuet-Higgins^16^.

Whilst these initial studies were limited to low impact velocities and small drops, Snyder^17^ and Jacobus^18^ took advantage of the 26 m tall drop facility at the Naval Postgraduate School in Monterey, CA, to test drops of various sizes falling at their terminal velocities. They found that a different jet-based sound-production mechanism existed for larger drops, and linked drop diameter to frequency for these drops. Jacobus also interestingly noted a direct linear correlation between the sound energy emitted by a drop impact and the temperature difference between the drop and the main body of water.

In 1992 Medwin^19^ confirmed these findings, noting the differing underwater sound-production mechanisms of four distinct drop-size ranges. Nystuen *et al*.^20^ further investigated the sound generated by the bubble-entrainment mechanism, whilst in 1996 the same author^21^ found that there were some instances where the impact sound was significant due to mid-sized drops travelling too fast to create a normal cavity, but not being large enough to produce sound via the jet-based mechanism first outlined by Snyder^17^. Attention also focussed on the detailed characteristics of the oscillating underwater air bubble, summarised in Leighton’s book on the subject^22^.

As has been shown, extensive literature exists on the underwater sound produced by drop impacts. However, there appears to be a distinct gap concerning the mechanism behind the airborne sound. Leighton^23^ suggests that the airborne sound is simply the underwater sound field propagating through the water-air interface, but no experimental study has been conducted to confirm this hypothesis. This study provides the first detailed investigation into the airborne sound produced by drop impacts, applying modern high-speed video and sound measurement techniques to the problem for the first time. This allows accurate causal links between the fluid dynamics of of the drop impact and the radiated sound field to be provided.

## Experimental Method

**Figure 1:**
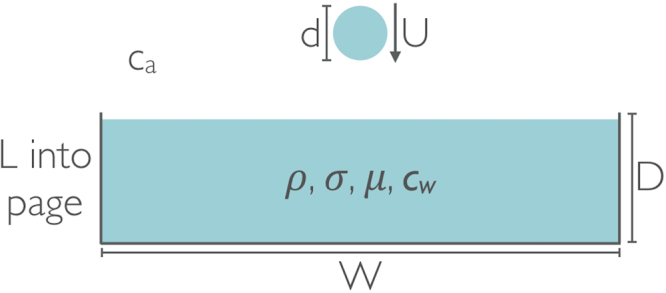
Schematic showing the parameters relevant to a typical drop impact.

The scenario considered, shown schematically in Fig. 1, involves a drop of liquid falling into a tank full of the same liquid. The dependent variable is the characteristic frequency, *f*, of the ‘plink’ sound produced by the drop impact, and as labelled in the figure the parameters affecting it are as follows: the drop’s equivalent spherical diameter, *d*, and impact velocity, *U*; the tank dimensions, *W* and *L*; the liquid’s depth, *D*, density, *ρ*, surface tension, *σ*, and dynamic viscosity, *μ*; and finally the speed of sound in both the air and the liquid, *c*_*a*_ and *c*_*w*_. Dimensional analysis shows that the frequency’s dependence on the tank dimensions (*W*, *L*, and *D*) and the speeds of sound (*c*_*a*_ and *c*_*w*_) is insignificant as the former can be chosen to be much greater than the drop diameter (*d*), and the latter are much larger than the drop’s impact velocity (*U*).

The mechanism of interest is the bubble entrainment described extensively in the literature on underwater sound production by drop impacts. Pumphrey *et al*.^11^ and Oquz and Prosperetti^14^ found that regular entrainment only occurred within a fixed range of impact velocities for a given drop size, and that no regular entrainment was observed for drop diameters smaller than 1 mm or larger than 5 mm. This indicated a need to control the drop’s diameter and impact velocity to ensure entrainment occurred regularly during experimentation.

Knowledge of the drop’s impact velocity was required, and a formula giving a drop’s impact speed when falling from a given height can be derived using a simple force balance on the falling drop. This formula, shown below in the form used by Pumphrey *et al*.^11^, gives the drop’s impact velocity, *U*, in terms of its drop height, *h*, and its terminal velocity, *V*_*T*_.

1 $$U={V}_{T}\sqrt{1-\exp (\,-\,\frac{2gh}{{V}_{T}^{2}})}$$

The drop’s terminal velocity, *V*_*T*_, depends on its drag coefficient which is non-trivial due to changes in shape caused by the interaction between the liquid and the air. To remedy this the method suggested by Beard^24^ was used, which combines dimensional analysis with fits to empirical data from Gunn and Kinzer^25^ to provide values of *V*_*T*_ for drops with equivalent spherical diameters in the range 1.07 mm ≤ *d* ≤ 7 mm. The resulting values agree to within ±0.5% of the experimental values given by Gunn and Kinzer. Snyder^17^ validated Beard’s method, a more detailed account of which can be found in Pruppacher and Klett’s volume^26^.

**Figure 2:**
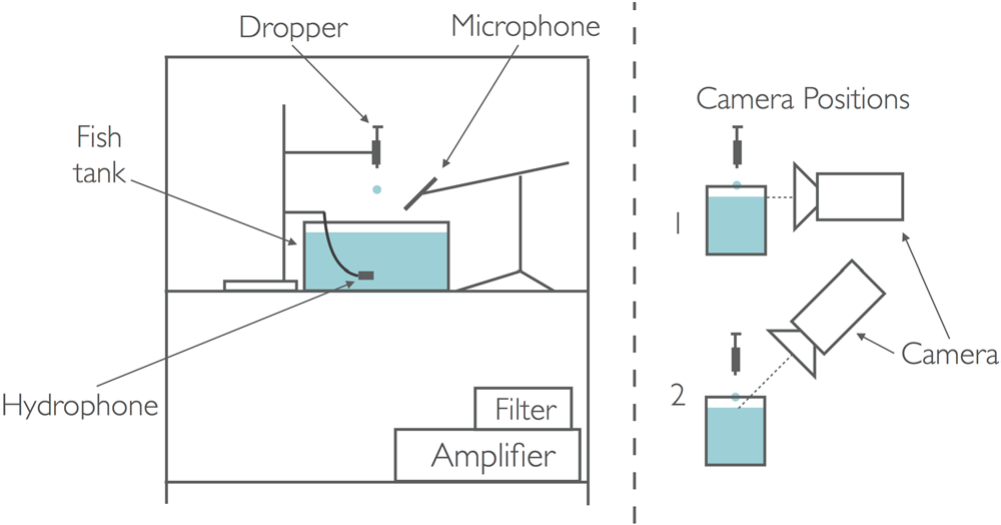
Schematic showing the experimental set-up used.

A schematic of the experimental set-up is shown in Fig. 2, and a full explanation can be found in the Supplementary Note published online. All data generated and analysed during the current study are available from the corresponding author on reasonable request.

## Results and Discussion

### A Typical Drop Impact

During the course of the experiments hundreds of drop impacts were recorded. They all exhibited some characteristic features which will be outlined here by studying the impact of a 4.0 mm diameter drop falling from a height of 86 mm (equating to an impact velocity of 1.29 ms^−1^).

**Figure 3:**
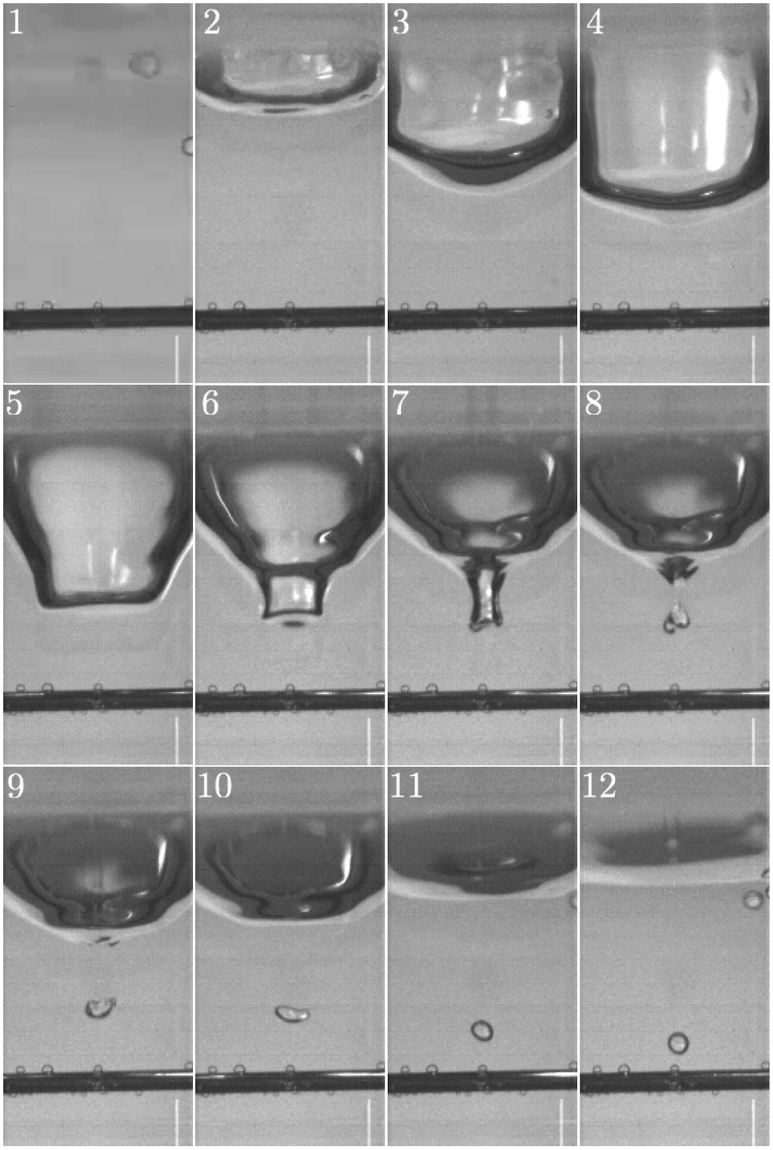
Key frames from the underwater high-speed video of a 4.0 mm diameter drop impacting the water surface at 1.29 ms^−1^. The cylindrical sizing rod has a measured diameter of 0.71 mm, and the white bar visible in the bottom right corner is a row of faulty pixels. Note that the elapsed time between each frame is not constant.

A high-speed video of this drop impact was taken at 30,000 fps with the camera positioned horizontally (position 1 in Fig. 2). Key frames from this video (available in full as Supplementary Video S1) are shown sequentially in Fig. 3, from which it is clear that the key features highlighted by Franz^9^ are accurate; the impact creates a cavity (frames 1 to 4), which then begins to recoil (frames 5 and 6); a small air bubble is then trapped under the water (frames 7 to 9) in a process referred to as bubble entrainment. The cylindrical rod visible in the lower portion of each frame had a measured diameter of 0.71 mm and was used to size the different features of the mechanism. Using this technique the maximum radius of the cavity was measured at 2.27 mm and entrainment was found to occur at a depth of 4.54 mm. The initial bubble, approximately spheroid in shape, had a major axis of 0.923 mm and a minor axis of 0.675 mm, with its final stabilised spherical diameter being 0.71 mm.

**Figure 4:**
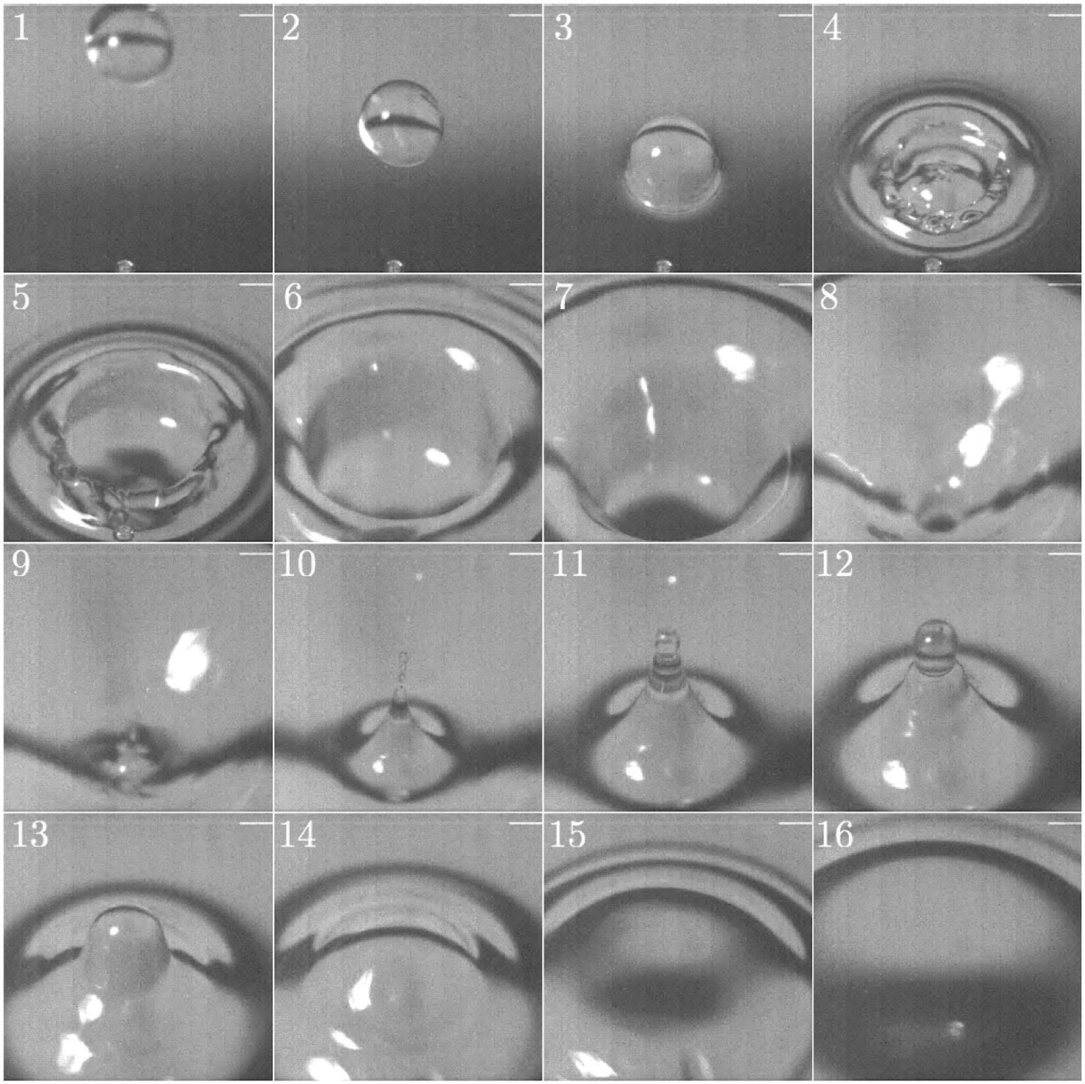
Key frames from the above water high-speed video of a 4.0 mm diameter drop impacting the water surface at 1.27 ms^−1^. Note that the elapsed time between each frame is not constant and the white bar visible in the top right corner is a row of faulty pixels.

Figure 4 shows frames from a video of a similar drop impact (available in full as Supplementary Video S2), this time looking down into the cavity from above (position 2 in Fig. 2). The experiment shown used the same drop diameter as that depicted in Fig. 3 and an impact velocity of 1.27 ms^−1^, slightly lower than that used in the underwater video but close enough for discussion of the general mechanism to be valid. On impact the drop deforms and causes a slight rise in the surrounding circle of fluid (frames 3 and 4). The cavity then forms (frames 5 to 7) before being seen to close up (frames 7 to 9), with this coinciding with the bubble entrainment seen in frames 5 to 9 of Fig. 3. A violent recoil of the water surface follows, with a rapidly rising column protruding from the centre of the cavity (frames 9 to 11). In some cases this column, or jet, breaks up due to capillary instability and forms small droplets which themselves impact the surface and produce cavities of their own. If no break up occurs, as seen in this case, the column recedes (frames 12 and 13), oscillating a few times (frames 14 and 15) before the flat water surface is restored.

**Figure 5:**
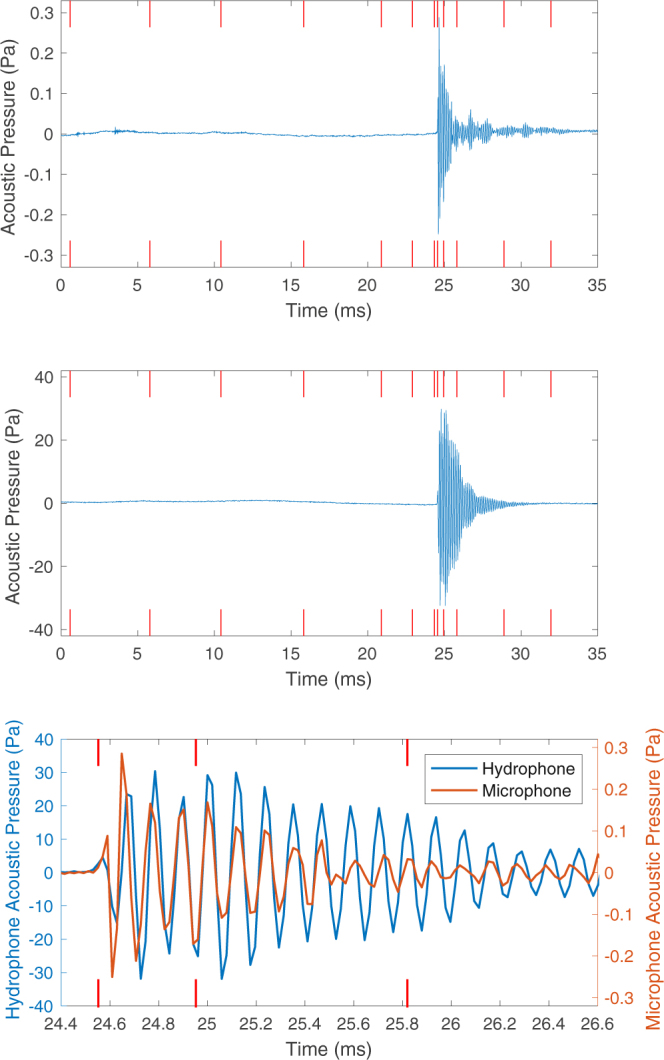
Airborne and underwater sound recorded when a 4.0 mm diameter drop impacted the surface at 1.29 ms^−1^. The upper and middle plots show the full airborne and underwater signals respectively. The lower plot shows an overlaid close-up of the decaying wave-packet. The red lines indicate the locations of the video frames shown in Fig. 3, with only frames 8, 9 and 10 being marked in the lower plot.

The upper two plots in Fig. 5 contain the airborne and underwater sounds recorded during the experiment shown in Fig. 3, with the red markers indicating the corresponding location of each of the twelve frames. These signals have been corrected for emission times and calibrated to give units of Pascals. The hydrophone signal (middle plot in Fig. 5) exhibits the same general features observed in previous literature on the underwater sound produced by drop impacts, with a delay followed by the initiation of a wave-packet. The slow decay rate of this wave packet seen here is due to reverberation in the fish tank, a phenomena investigated in more detail later. The airborne sound signature (upper plot in Fig. 5), which is of primary interest in this investigation, exhibits the same delay and wave-packet structure as the underwater signal, suggesting that the mechanism behind the two sounds may be related. In both signals the wave packet is the key feature which will be looked at in more detail.

The lower plot in Fig. 5 shows a zoomed-in view of the decaying wave-packet, with the red marks indicating the location of frames 8, 9 and 10 in Fig. 3. The microphone and hydrophone signatures have also been overlaid to ease comparison. The two signals are extremely well aligned, with the small initial offset most likely a result of experimental errors in measuring the distance from each recording device to the impact site which effects the emission time correction. The sound is initiated at frame 8 in Fig. 3, which is exactly the point at which the bubble breaks away from the cavity. This confirms that the bubble entrainment mechanism is the key sound producing feature both above and below water, and so this wave packet will herein be called the bubble sound.

As no further sound features occur after this bubble sound it is concluded that none of the above water flow features seen in Fig. 4, other than those directly associated with the initial pinch off of the bottom of the cavity, contribute to the characteristic ‘plink’ sound that is of interest. This is a remarkable result given the amount of unsteady motion seen in frames 9 to 16 of Fig. 4. However, the lack of sound generation by these features can be explained. The first potential surface sound source are the ripples propagating radially outwards from the impact site, first seen in frame 4. These are capillary waves with a wavelength of around 2 mm, and using the dispersion relationship derived by Klemens^27^ their velocity is found to be of the order of 0.5 ms^−1^. As this is less than the speed of sound in air these surface disturbances cannot transmit acoustic pressure waves. The second possible sound generator is the oscillation of the water droplets thrown into the air by the recoiling surface. As water is essentially incompressible any oscillations of these droplets will be shape oscillations, rather than volume oscillations. Such compact oscillations represent a quadrupole acoustic source, and hence the water drops are extremely inefficient acoustic generators.

Comparing the two signals in Fig. 5 the most obvious difference is the more rapid decay of the airborne sound. This is due to underwater reverberations in the fish tank, an issue investigated later. The first oscillation in the microphone signal appears to be compressed compared to the underwater equivalent, and this was a consistent feature observed over multiple drop impacts. The reason for this initial compression is unknown and may be an interesting area of further research.

Frequency analysis of the two bubble signals reveals that both have the same dominant frequency at 8.66 kHz. For well over a hundred drop impacts the dominant frequency of the bubble sound above and below the water was found to agree to roughly within the resolution (400 Hz) of the discrete-time Fourier transform (DFT) used. This again suggests that the underlying mechanism driving each sound system is the same.

**Figure 6:**
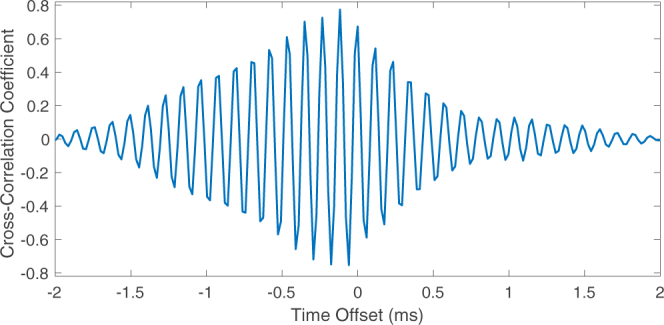
Statistical cross-correlation of the airborne and underwater bubble sounds presented in the lower plot of Fig. 5.

One way to mathematically demonstrate the similarity of two signals is through use of statistical correlation analysis. Figure 6 shows a plot of the cross-correlation coefficient for the two signals in the lower plot of Fig. 5. A maximum cross-correlation coefficient of 0.777 was reached at a time offset of −0.12 ms. This offset is small and within the range of error associated with the distance measurements used for the emission time correction. Over the course of hundreds of recorded drop impacts the maximum cross-correlation coefficient between the airborne and underwater bubble sounds varied between around 0.5 and 0.8, with these large maximum values mathematically confirming the correlation between the two signals.

### Preventing Entrainment

**Figure 7:**
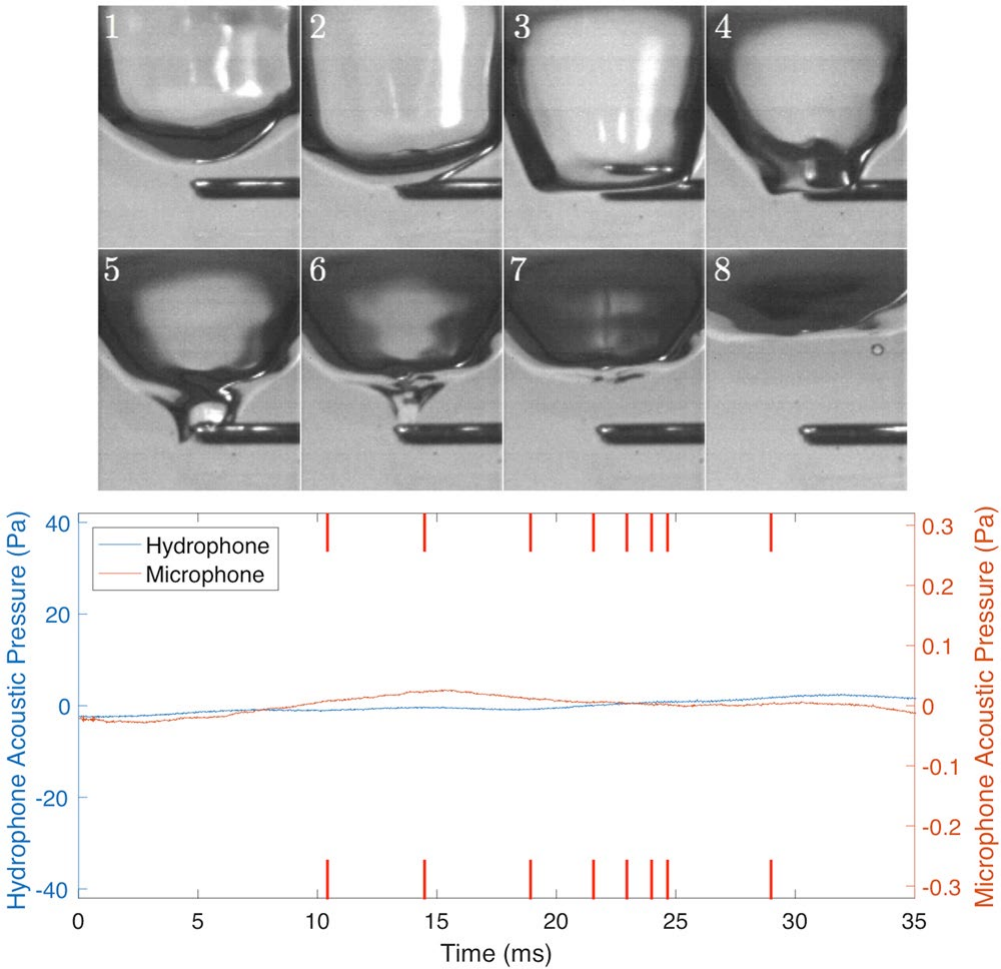
Select frames showing entrainment being prevented by the rod, with the recorded airborne and underwater sound signals shown below. The red lines on the audio plot indicate the approximate locations of the video frames above.

If the entrained bubble is in fact responsible for the ‘plink’ sound then suppressing the bubble should suppress the sound. This was achieved in two different ways: firstly by using the sizing rod, and secondly by introducing a surfactant. Figure 7 shows frames from a video of the former case (full video available as Supplementary Video S3), alongside the recorded above- and below-water sound signals. The video frames show the usual cavity forming before the rod prevents entrainment of the underwater air bubble. The recorded sound signals demonstrate that no sound is produced when bubble entrainment is prevented, confirming the earlier conclusion that this bubble-entrainment mechanism underpins production of the characteristic ‘plink’ sound.

A similar result was achieved using the second method, with washing-up liquid used as the surfactant. A video of the resulting drop impact is available as Supplementary Video S4. Bubble entrainment was prevented and no sound was produced, again confirming that the bubble-entrainment mechanism underpins the characteristic ‘plink’ sound that is of interest. Interestingly, this second result also suggests that small changes in surface tension have an effect on bubble entrainment and so this addition of a small amount of washing up liquid to the body of water may represent a simple and novel way to alleviate the annoying noise of a leaking tap or water dripping from a roof into a bucket.

### Frequency Comparison

In his 1933 paper Minnaert^6^ derived the following formula for the natural oscillation frequency of a submerged gas bubble by using energy arguments:

2 $$f=\frac{1}{2\pi a}\sqrt{\frac{3\gamma {P}_{o}}{\rho }}$$

In this formula *f* is the natural frequency, *a* is the bubble radius, *γ* is the ratio of specific heats for the gas inside the bubble, *P*_*o*_ is the static pressure on the exterior surface of the bubble, and *ρ* is the density of the liquid. Using the sizing rod as depicted in Fig. 3 the initial bubble dimensions could be measured from the videos of a number of drop impacts and used in the above equation to calculate its theoretical oscillation frequency. This can then be compared to the frequency of the bubble sound recorded experimentally. The results for five tests are presented in Table 1.

**Table 1:** Comparing the theoretical (Minnaert) and experimentally recorded bubble frequencies.

Test Number	Recorded Frequency (kHz)	Minnaert Frequency (kHz)	Percentage Difference (%)
1	7.90	7.57	−4.1
2	8.66	7.90	−8.8
3	8.41	7.71	−8.3
4	11.72	15.11	+29.0
5	12.23	16.97	+38.8

The first three tests, each exhibiting a recorded frequency of around 8 kHz and a relatively large entrained bubble, show remarkably good agreement between the theoretical and experimental frequencies. Errors of less than ±10% are extremely small, especially given the inaccuracies associated with measuring bubble sizes from two-dimensional video frames. The errors may also be down to the fact that Minnaert’s theory assumes that the bubble is spherical and is oscillating in an infinite domain, neither of which is true in this case due to the distortion of the entrained bubble and the presence of the water surface. Strasberg^28^ developed correction factors that may be applied to Minnaert’s formula for non-spherical bubbles and those close to a flat surface, both of which act to increase the natural frequency predicted by Minnaert and so would reduce the errors observed here. Accurate modification of the predicted frequency in this case is not possible due to the complex initial shape of the entrained bubble and the curved nature of the water surface at the bottom of the cavity (see frame 8 in Fig. 3). Even without any correction being applied the small errors observed between the experimentally measured and theoretically determined bubble frequencies are remarkable and provide further proof that it is volume oscillations of the entrained bubble that drive both the above- and below-water sound fields.

Tests 4 and 5 in Table 1 exhibit higher recorded frequencies, and the differences between the experimental and theoretical frequencies in these cases is significant. The bubble size in these tests was much smaller than in the cases where there was good agreement, despite each of the experiments being conducted using the same sized drops falling with the same impact velocity, highlighting issues of experimental reliability. It may be that these smaller bubbles oscillate in a different way to that investigated by Minnaert, or that a different mechanism is driving the sound field in these instances. A simpler explanation could be that the entrained spheroid bubble was being viewed end on, leading to inaccurate measurement of the bubble size and hence large errors in the theoretically calculated frequencies. However, when the ‘settled’ bubble size was used, which should give the true equivalent spherical diameter of the initial bubble as its volume is constant, the Minnaert frequency rose to over 20 kHz for both tests 4 and 5, discounting this simple explanation for the discrepancy.

Despite the larger errors in tests 4 and 5, it is clear from tests 1, 2 and 3 that a regime does exist in which the experimentally measured frequency of the bubble sound aligns with the theoretically predicted natural oscillation frequency of a similarly sized underwater bubble. This is further proof that volume oscillations of the entrained bubble are driving both the above and below water sound fields.

### High-Performance Video Observations

Towards the end of the investigation ultra high-speed, high-resolution videos of the bubble-entrainment mechanism were captured using a Vision Research V2512 Mono camera and an enhanced LED lighting set-up. The improved performance allowed for video capture at a frame rate of 75,000 fps and a resolution of 512 × 512 pixels. Unfortunately cooling fans in the LED lights led to slight contamination of the acoustic signatures, with the plot corresponding to Fig. 5 not being as clear. This data has therefore been relegated to the online supplementary material, where it is presented as Supplementary Videos S5 and S6 and Fig. S2.

In the video of the drop impact (Supplementary Video S5) volume oscillations of the entrained bubble can be directly observed and, despite the contamination, qualitatively matched up to the recorded bubble sound signal (with the aid of Supplementary Video S6). The pressure field that arises due to a pulsating sphere (a good model for the oscillations of the entrained bubble) is proportional to the acceleration of the volume within the sphere,

3 $$p \sim \frac{{d}^{2}V}{d{t}^{2}}$$

The pinch-off as the bubble is entrained (see frame 8 of Fig. 3) is the driving force behind this volume acceleration, and in the high-performance videos the bubble could be seen to expand immediately following pinch-off. This initial expansion means that the volume inside the bubble is increasing, and hence the volume acceleration is initially positive. This should result in an initial rise in the pressure field and Fig. 5 shows that this is the case. Following this initial expansion only a small number of volume pulsations can be seen, which matches up with the number of oscillations seen in the recorded airborne bubble sound signal.

All of these observations of the high-performance video confirm beyond doubt that volume oscillations of the entrained bubble drive the above and below water sound fields. This is the first time oscillations of the entrained bubble have been directly observed and aligned with the resulting audio signals.

### Sound Propagation Through The Water Surface

Given that oscillations of the entrained bubble drive both sound fields, the question turns to the mechanism by which this occurs. Underwater, pressure waves may travel unimpeded from the oscillating bubble to the hydrophone, but this does not explain how the oscillating bubble is driving the sound field heard above the water. One possible explanation is that the sound radiated underwater simply propagates through the water-air interface. Leighton^23^ puts forward this hypothesis supported by some simple feasibility calculations. Such calculations indicate an attenuation of around 70 dB across the interface, which is significant and, crucially, much greater than the attenuation levels recorded during this investigation. Comparing the signals in Fig. 5 the acoustic pressure at the second peak in each signal (as after this reverberation has significant effects) is around 24 Pa below the water and 0.28 Pa above. This equates to an attenuation of around 39 dB, 30 dB lower than that predicted by the simple propagation calculations. The recorded attenuation level was consistently found to be around 40 dB throughout these experiments and the size of the discrepancy between the theoretical and experimental attenuation factors casts serious doubt over the idea that the airborne sound field is simply the underwater sound propagating through the water-air interface.

### The Effect of Reverberation

Throughout the experiments reverberation within the fish tank was expected to have had some effect on the results. To investigate these effects the tank’s inner walls were lined with 11 mm thick MDF wood to remove some reverberation. Recordings of drop impact sounds were taken with and without the wood in place and compared to observe the effects of reverberation. To ensure a consistent effect was being observed several drop impacts were recorded and the resultant bubble signals averaged. Each result presented in this section used a 4 mm diameter drop with an impact velocity of 1.36 ms^−1^.

**Figure 8:**
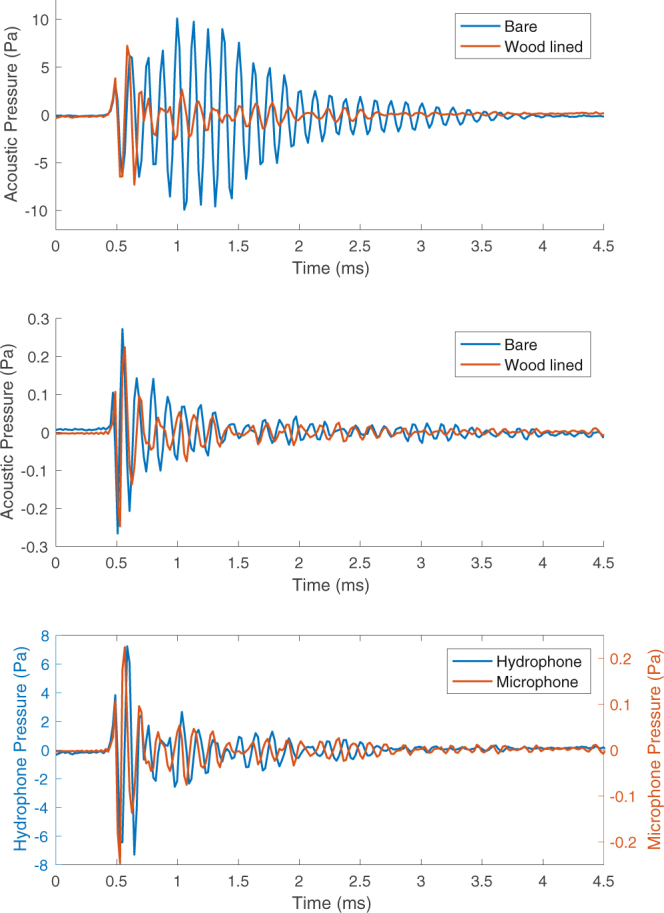
Results of the reverberation investigation. The upper and middle plots show the averaged bubble signal recorded in the bare and wood lined tanks below- and above-water respectively. The lower plot shows an overlaid plot of the averaged underwater and airborne bubble signals recorded in the wood lined tank.

The upper plot in Fig. 8 shows the averaged underwater bubble signals recorded in the bare (blue) and wood-lined (orange) tanks overlaid to ease comparison. It is clear from this Figure that reverberation has significantly altered the recorded underwater sound signal. The initial oscillations in both cases are nearly identical, but in the bare tank the amplitude then grows whilst in the wood-lined tank it decays. This amplitude growth must therefore be caused by the interaction of multiple reflections bouncing off the tank walls, with these reflections diminished by the wood lining.

A similar plot for the airborne sound is shown in the middle of Fig. 8, which demonstrates that reverberation has a much smaller effect on this signal. The minor changes observed are probably due to natural variation in the results. What is of most interest is the difference between the effects of reverberation on the airborne and underwater sound fields. If the underwater sound field was simply propagating through the water-air interface to produce the airborne ‘plink’ sound then the two signals would be identical in every case, as any underwater reverberation would also propagate through the interface and be recorded above the water. The upper and middle plots in Fig. 8 clearly show that removing reverberation dramatically changes the underwater signal but has no effect on the airborne signal. This fact and the discrepancy between the theoretical and experimentally measured attenuations clearly demonstrates that the characteristic airborne ‘plink’ sound is not simply the underwater sound field propagating through the water-air interface.

One final point of interest is how well the above and below water sound signals align when reverberation is removed. The lower plot in Fig. 8 shows the averaged above and below water bubble signals in the wood lined tank overlaid. The similarity is striking but not surprising given that both sound fields are being driven by the same mechanism.

### Proposed Theory for Sound Generation

**Figure 9:**
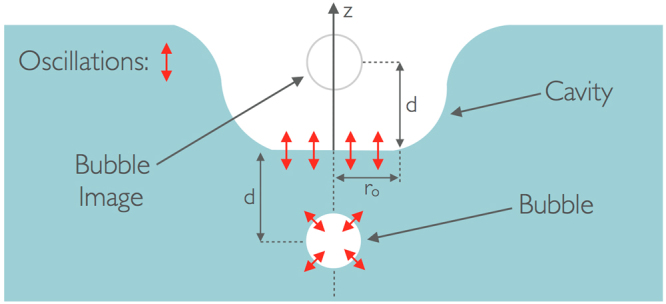
Schematic showing the entrained bubble oscillations driving oscillations of the water surface. Oscillations are indicated by double-headed red arrows.

Having demonstrated that the above water sound field is not simply the underwater field propagating through the water-air interface, an alternative mechanism by which the entrained bubble may drive the airborne sound field has been developed. The hypothesis is that as soon as the bubble is released underwater its oscillation induces an oscillation of the water surface itself at the same frequency. This occurs because, in the vicinity of the bubble, the flow may be considered to be potential as water is nearly incompressible and the oscillatory motion is extremely fast. Thus when the bubble is close to the free surface, just after entrainment, its oscillations push and pull on the water surface, driving oscillations at the same frequency. This idea is shown schematically in Fig. 9.

The portion of the water surface closest to the bubble will be excited most strongly, and in this case that is the bottom of the cavity as indicated by the red arrows in the figure. Because the cavity depth is small compared to the acoustic wavelength, the sound radiated from this segment of the surface can be modelled as a piston, of radius *r*_*o*_, in an infinite baffle for which the pressure perturbation (denoted by a prime to differentiate it from the mean) along the *z* axis is given by Blackstock^29^ to be

4 $$|p^{\prime} (z,\,f)|=\frac{{r}_{o}{\rho }_{a}{c}_{a}{u}_{o}}{z}\frac{k{r}_{o}}{2}$$

where *ρ*_*a*_ is the air density, *c*_*a*_ is the speed of sound in air, *u*_*o*_ is the velocity of the ‘piston’, and *k* is the wavenumber. In our case the piston velocity, *u*_*o*_, can be estimated using the velocity perturbation arising from the pulsating bubble. By using an image source as shown in Fig. 9, the underwater pressure perturbation generated by the bubble along a line running through its centre perpendicular to the water surface is given by

5 $${p^{\prime} }_{w}(r,\,f,\,t)=\frac{A}{r}\exp (i(\omega t-\frac{r}{{c}_{w}}))\times \frac{2\omega d}{{c}_{w}}$$

where *r* is the distance from the bubble, *ω* is the radian frequency, *t* is the time, *c*_*w*_ is the speed of sound in water, *d* is the distance from the bubble’s centre to the water surface, and *A* is a constant. From this pressure perturbation the velocity perturbation can be found using the momentum equation, and for $r\ll 1$ it can be shown that

6 $${v^{\prime} }_{w}(r,\,f,\,t)\approx \frac{2dA}{i{\rho }_{w}{c}_{w}{r}^{2}}\exp (i(\omega t-\frac{r}{{c}_{w}}))$$

Therefore the magnitude of the velocity perturbation at the water surface, a distance *d* from the bubble, is

7 $$|{v^{\prime} }_{w}(d,\,f,\,t)|\approx {u}_{o}\approx \frac{2dA}{{\rho }_{w}{c}_{w}{d}^{2}}$$

Substituting this into Equation 4 results in the following expression for the magnitude of the sound pressure field in air radiated by the oscillating water surface:

8 $$|{p^{\prime} }_{a}(z,\,f)|=\frac{{r}_{o}{\rho }_{a}{c}_{a}}{z}\frac{k{r}_{o}}{2}\frac{2dA}{{\rho }_{w}{c}_{w}{d}^{2}}$$

To compare the above and below water sound fields the ratio of the microphone and hydrophone pressures must be found. For the hydrophone the pressure field is given by Equation 5 with *r* = *R*, the distance of the hydrophone from the bubble. For the microphone the pressure field is given by Equation 8 with *z* = *z*_*o*_, the distance of the microphone from the impact site. The ratio of the above and below water sound fields can thus be calculated to be

9 $$\frac{|{p^{\prime} }_{a}({z}_{o},\,f)|}{|{p^{\prime} }_{w}(R,\,f)|}=\frac{1}{2}\frac{{\rho }_{a}}{{\rho }_{w}}\frac{{r}_{o}^{2}}{{d}^{2}}\frac{R}{{z}_{o}}$$

During these experiments the distances of the microphone and hydrophone from the impact site were approximately equal, so *R* ~ *z*_*o*_. Observations during the experiment show that the radius of the bottom of the cavity is approximately three times the distance of the surface from the bubble, and so *r*_*o*_ ~ *3d*, or ${(\frac{{r}_{o}}{d})}^{2}\, \sim \,10$. Substituting these approximations into the expression for the ratio of the above and below water pressure fields, along with values for the densities of water and air, gives

10 $$\frac{|{p^{\prime} }_{a}|}{|{p^{\prime} }_{w}|} \sim \frac{1}{2}\times \frac{1.2}{1000}\times 10$$

which equates to an attenuation of ~44 dB. Throughout these experiments the attenuation between the above- and below-water sound signals was consistently found to be around 40 dB. This simple model predicts a remarkably similar level of attenuation, supporting this new hypothesis for how the underwater bubble oscillations produce audible sound above water. This demonstrates that the entrained bubble’s close proximity to the bottom of the cavity is a crucial factor in the production of the characteristic loud ‘plink’.

## Conclusions

The impact of a drop of liquid falling on a free surface creates a very rich sequence of dynamics that involves: the initial impact, capillary waves on the surface, a cavity due to the inertia of the drop, a recoil of the cavity due to surface tension, an entrapped air bubble, the recoiling surface forming a jet that disintegrates into a liquid drop above the air surface. This investigation has used modern high-speed video techniques for the first time, synchronised with microphone recordings in air and hydrophone in water to demonstrate that the oscillation of an entrapped air bubble is the key driver for both the underwater sound and the characteristic airborne ‘plink’. All the other features are effectively silent. However, the airborne sound is not simply the underwater sound field propagating through the water surface, as had been previously thought. In order for the ‘plink’ to be significant, the oscillating bubble needs to be in close proximity beneath the air cavity created by the inertia of the drop impact. The bubble then drives the bottom of the cavity like a piston in a baffle. The predictions based on this model are found to agree well with all the experimental data collected.

## Electronic supplementary material

Supplementary Information

Supplementary Video S1

Supplementary Video S2

Supplementary Video S3

Supplementary Video S4

Supplementary Video S5

Supplementary Video S6
