# Supplementary material for: The Sound Produced by a Dripping Tap is Driven by Resonant Oscillations of an Entrapped Air Bubble
Source: Sci Rep. 2018 Jun 22;8:9515. doi: 10.1038/s41598-018-27913-0 (PMC6014985; doi:10.1038/s41598-018-27913-0)
Supplement: Supplementary file 1 — Supplementary Information [file 41598_2018_27913_MOESM1_ESM.pdf]

The Sound Produced by a Dripping Tap is Driven by Resonant Oscillations of an Entrapped Air Bubble
Supplementary Material

Samuel Phillips, Anurag Agarwal and Peter Jordan

Supplementary Note on Experimental Set-up

Figure S1 shows a schematic of the experimental set-up used during the experiments. Drops were produced and fell a specified distance into a fish tank filled with water. The resultant sound was recorded above and below water using a microphone and hydrophone respectively, whose positions are shown in the schematic. The recorded signals were filtered and amplified before being logged using a National Instruments data logger connected to a computer running Matlab. The drop impact was also filmed using a high-speed camera. Tap water was used throughout allowing for direct application of the results to the scenario of a dripping tap. All experiments were carried out in an anechoic chamber.

The following subsections outline key aspects of the experimental set-up in more detail.

Drop Production

A key element of the experiment was reliable and accurate production of different sized water drops. Several techniques are found in the literature; Pumphrey et al. [1] used a syringe to force fluid through hypodermic needles of various diameters, whereas Kurgan [2], Snyder [3] and Jacobus [4] all made use of Eppendorf digital pipettes. A syringe and hypodermic-tube set-up similar to that described by Pumphrey et al. [1] was used here. The tube diameter was 1.82 mm, and it was found to produce drops with an equivalent spherical diameter of 4 mm. The method of calibration was to measure the volume of liquid produced by 100 drops and use this to calculate the volume, and hence equivalent diameter, of a single drop, assuming each of the drops produced was identical. This was carried out a number of times to improve accuracy, with the final error judged to be no more than ±5%. This drop size exhibited regular entrainment behaviour within a small range of impact velocities, improving the reliability of the experiment.

Sound Acquisition

The positioning of the two sound-acquisition devices is shown in Figure S1, with the hydrophone placed just below the drop impact site, and the microphone pointed towards the impact site just outside the splash zone, both at a distance of around 80 mm from the impact site. A LinearX systems M51 microphone

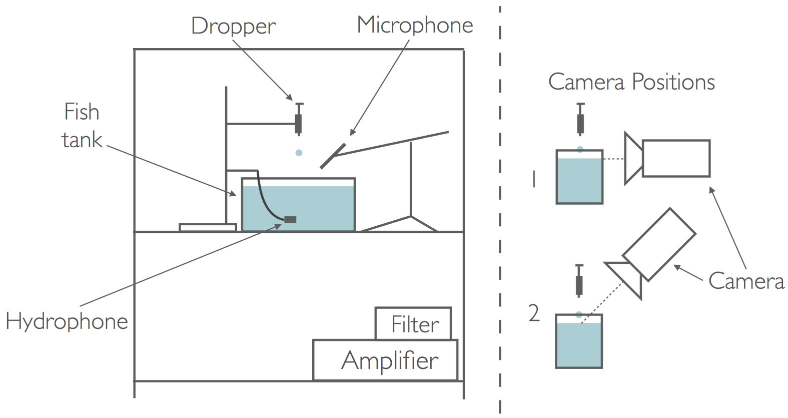

Figure S1: Schematic of the experimental set-up and camera positioning.

was used, which exhibited a flat frequency response (±10 dB) in the range 0 Hz to 30 kHz, with a quoted sensitivity of 13.2 mV/Pa. The signal was sent through a low-pass filter to prevent aliasing and remove unwanted high frequency noise, and then an amplifier of gain 20. The hydrophone was a Brüel and Kjær type 8103, with a quoted voltage sensitivity of 25.9 µV/Pa and a flat frequency response (±1 dB) in the range 4 kHz to 200 kHz. This signal was passed through the same manufacturer's type 2635 charge amplifier. Knowledge of the microphone and hydrophone sensitivities and amplifier settings allowed the recorded voltage signals to be converted into Pascals. Both signals were sampled using a National Instruments NI-9234 synchronous four channel data logger, which enabled simultaneous readings to be taken at sampling frequencies of up to 51.2 kHz.

It was important to account for the time taken for the sound to travel from the impact site to the recording devices. In water sound takes around 50 µs to travel the 80 mm to the hydrophone, whereas travelling the same distance in air to the microphone takes around 240 µs. The difference in these two values highlights the importance of correcting the time scales of the recorded signals to align with the fluid-mechanical sound-generating events. This was done as part of the post-processing of the data, using standard values for the speeds of sound in air and water as well as the measured distances from the hydrophone and microphone to the impact site.

High-speed Video Recording

A FASTCAM-ultima APX was used to record the high-speed videos, shooting in black and white up to a maximum of 120,000 frames-per-second (fps). Higher frame rates required more powerful lighting, and also resulted in significantly reduced resolution (down to 128 x 16 pixels at the maximum frame rate, from 1024 x 1024 pixels at 2,000 fps). A frame rate of 30,000 fps was used as standard which is substantially higher than any used in previous work on drop impacts. A Nikon micro-NIKKOR 55 mm f/2.8 lens was used to focus on the impact site. The two camera positions adopted are shown in Figure S1, with the first looking horizontally at the drop-impact and bubble-entrainment mechanisms and the second looking down into the cavity formed by the drop impact. The videos were recorded using a PC running PFV - Photron FASTCAM viewer and processed using Quicktime and Matlab.

Lighting was provided by two 600 W floodlights positioned behind the camera, approximately 20° either side. To provide a bright backdrop the exterior walls of the fish tank were lined with white paper.

Synchronisation between the audio and video recordings was achieved using the camera's General Out connection which sent a steady voltage signal when the camera was recording. This was sampled by the same data logger described previously. The delay in the pulse was quoted in the camera's documentation as 55 ns, meaning this synchronisation technique was sufficiently accurate for the purposes of these experiments.

Sizing information about various flow features was obtained from the videos by placing a cylindrical rod, of known diameter, in the frame far enough below the impact site to ensure it did not interfere with the entrainment mechanism. A calibration of pixels per mm could then be obtained, enabling the size of the entrained bubble and other flow features to be determined. This sizing rod was also used in later experiments to disturb the entrainment mechanism to observe the effect on the sound field.

One issue during the experiments was a lack of consistency in the drops producing the characteristic 'plink' sound, even within the 'regular entrainment' region. On several occasions the drops went from consistently producing the characteristic 'plink' to producing no sound within a matter of hours, with no change to the experimental set-up. There are two hypotheses regarding this lack of consistency.

Firstly, it may have been due to small particulates in the water disturbing the bubble entrainment mechanism. This hypothesis is supported by the fact that drops produced the characteristic 'plink' sound more reliably if the water was left overnight, allowing particulates to sink to the bottom and so not affect the impact site. Secondly, it may have been due to variations in surface tension due to oil and dirt passing from hands, the hydrophone, and the tank walls into the water. To determine whether either of these hypotheses is correct would require a more detailed study into the factors effecting bubble entrainment.

Despite being a source of difficulty during experimentation, this issue does not impact the results and conclusions presented in this paper. It does not affect the mechanism of sound production once a bubble has been entrained, which is the focal point of this investigation.

Supplementary Experimental Data

The videos that form the basis for much of the discussion in the paper are presented as Supplementary Videos S1 - S4, uploaded separately online. Supplementary Video S1 shows a typical drop impact with

the camera located at position 1 in Figure S1, while Supplementary Video S2 shows a very similar drop impact with the camera located at position 2. Supplementary Video S3 shows the bubble being perturbed by a rod, with the resulting audio signal shown in Figure 7 of the main paper. Supplementary Video S4 shows a drop impact when a surfactant (washing up liquid) had been added to the surface of the water.

The final items of Supplementary Material concern an equivalent to Figures 4 and 5 in the main paper when the high-performance video camera and LED lighting rig were used. This is presented as Supplementary Figure S2, with the corresponding video footage available as Supplementary Video S5. The audio signals have been contaminated by vibrations caused by fans within the LED lights used. Despite this the same decaying wave-packet structure can be observed. The oscillations seen in the close-up view of this wave-packet can be qualitatively matched to the observable volume oscillations in the video with the aid of Supplementary Video S6, in which the video frames and audio signals have been positioned alongside one another.

References

[1] H. C. Pumphrey, L. A. Crum, and L. Bjorno. Underwater sound produced by individual drop impacts and rainfall. *J. Acoust. Soc. Am.*, 85:1518–1526, 1989.

[2] A. Kurgan. Underwater sound radiated by impacts and bubbles created by rainfall. Master's thesis, Naval Postgraduate School, Monterey, CA, 93943, 1989.

[3] D. E. Snyder. Characteristics of sound radiation from large raindrops. Master's thesis, Naval Postgraduate School, Monterey, CA, 93943, 1990.

[4] P. W. Jacobus. Underwater sound radiation from large raindrops. Master's thesis, Naval Postgraduate School, Monterey, CA, 93943, 1991.

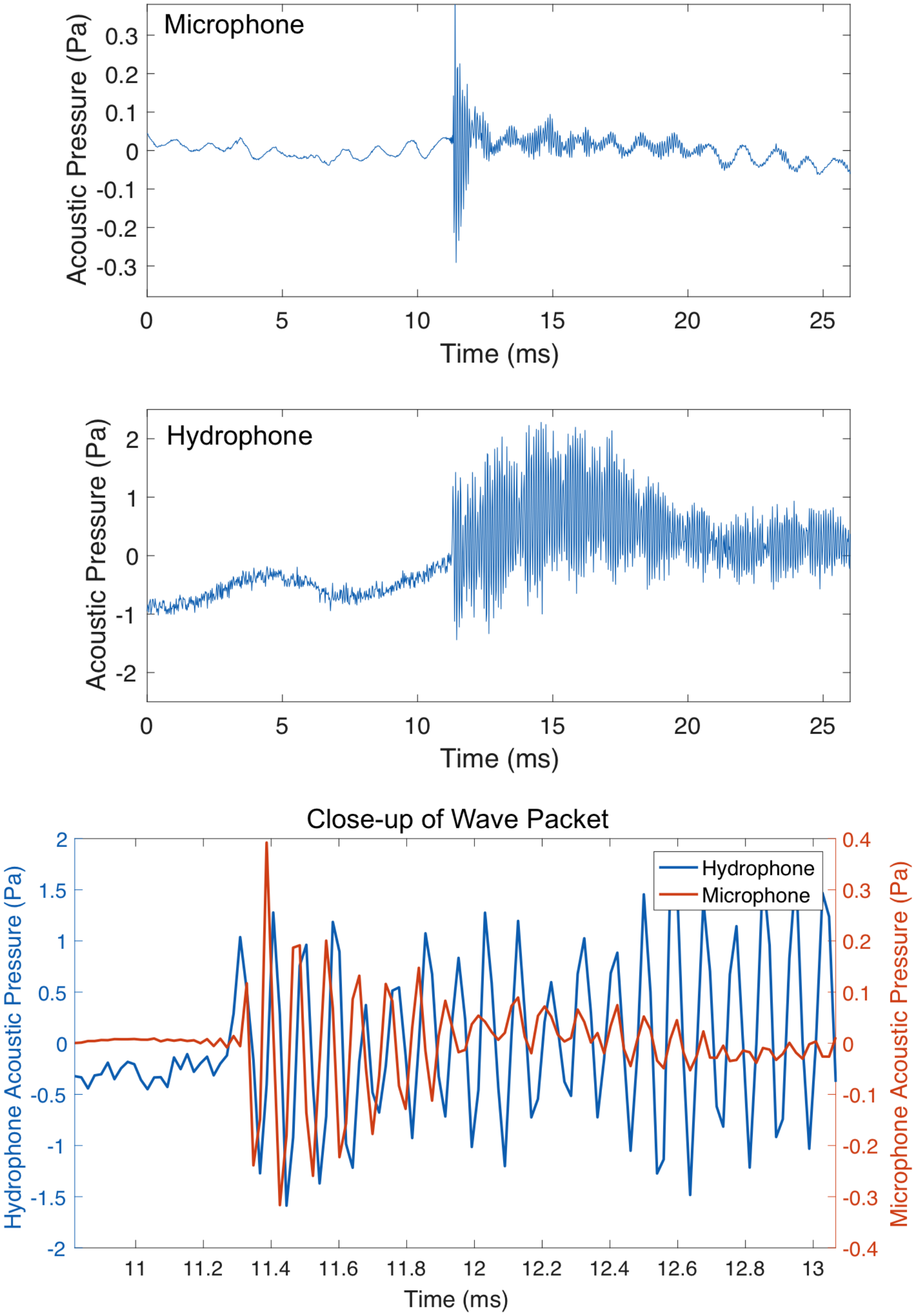

Figure S2: Airborne and underwater sound recorded when a 2.673 mm diameter drop impacted the surface at 1.744 ms^{-1}. The upper and middle plots show the full airborne and underwater signals respectively. The lower plot shows an overlaid close-up of the decaying wave-packet. A video of this drop impact is available as Supplementary Video S5.
